# Complete Genome Sequence of Lytic Bacteriophage LZ35 Infecting *Acinetobacter baumannii* Isolates

**DOI:** 10.1128/genomeA.01104-16

**Published:** 2016-11-17

**Authors:** Zhonghe Guo, Honglan Huang, Xiaolin Wu, Yuchong Hao, Yanbo Sun

**Affiliations:** Department of Pathogen Biology, College of Basic Medical Sciences, Jilin University, Changchun, People’s Republic of China

## Abstract

*Acinetobacter baumannii* is a Gram-negative opportunistic pathogen that is frequently associated with nosocomial infections. Bacteriophages infecting *A. baumannii* can be used as effective agents to control these infections. Here, we announce the complete genome sequence of the lytic bacteriophage LZ35 infecting *A. baumannii* isolates.

## GENOME ANNOUNCEMENT

*Acinetobacter baumannii*, a nonfermentative, aerobic, Gram-negative bacillus, is a significant cause of opportunistic infections, such as ventilator-associated pneumonia, urinary tract infections, and bacteremia in intensive care units (1). Due to the increasing resistance to many classes of antibiotics among *A. baumannii* strains (2, 3), phage therapy (4, 5), an application of bacteriophages (phages) as an antibacterial agent, may be an alternative potential for controlling *A. baumannii* infection. Here, we present the complete genome sequence of lytic bacteriophage LZ35, which is infectious to *A. baumannii*.

Bacteriophage LZ35 was isolated from sewage effluents from the Jilin University First-Affiliated Hospital. The morphology of LZ35 was determined using transmission electron microscopy and demonstrated a 47-nm head diameter and a 56-nm-long contractile tail, which are characteristics of members of the family *Myoviridae*. Phage DNA was sequenced in an Illumina MiSeq 250-bp paired-end run with a 546-bp insert library at Suzhou Genewiz Biological Technology Co., Ltd. (Suzhou, People’s Republic of China). After trimming with Trimmomatic (http://usadellab.org/cms/?page=trimmomatic) and removal of low-quality ends, 3,592,478 reads comprising a mean coverage of 17,647-fold were *de-novo* assembled using SSPACE (http://baseclear.com/genomics/bioinformatics/basetools/SSPACE) and GapFiller (http://baseclear.com/genomics/bioinformatics/basetools/gapfiller). Gene prediction and annotation of the phage LZ35 genome was performed using the RAST server (6). Phage LZ35 contained a 44,885-bp double-stranded DNA and a G+C content of 37.95%, with no tRNAs identified. A total of 83 coding sequences were identified. The analysis of the LZ35 genomic sequence reveals 78% and 77% matches with 97% and 99% identity to the *Acinetobacter* phage IME-AB2 (accession no. JX976549.1) (7) and *Acinetobacter* phage YMC-13-01-C62 (accession no. KJ817802.1) genomes, respectively.

The genomic data of lytic phage LZ35 will prove useful for comparative studies and for the future investigation of LZ35 acting as a potential alternative for controlling *A. baumannii* infection.

### Accession number(s).

The complete genome sequence of *A. baumannii* phage LZ35 has been submitted to the GenBank database under the accession number KU510289. The version described in this paper is the first version, KU510289.1.
